# Protocol for the analysis of hematopoietic lineages in the whole kidney marrow of adult zebrafish

**DOI:** 10.1016/j.xpro.2023.102810

**Published:** 2024-01-22

**Authors:** Christopher B. Mahony, Rui Monteiro

**Affiliations:** 1 Institute of Cancer and Genomic Sciences, College of Medical and Dental Sciences, University of Birmingham, Birmingham, UK

**Keywords:** Cell isolation, Single Cell, Flow Cytometry, Model Organisms, Gene Expression

## Abstract

The whole kidney marrow (WKM) is the site for hematopoiesis in the adult zebrafish. Here, we present a protocol for analyzing hematopoietic lineages in the WKM of adult zebrafish. We describe steps for the isolation of hematopoietic cells from the WKM, the downstream analysis of total marrow cellularity, and analysis of cell populations by flow cytometry. We then detail procedures for May-Grünwald-Giemsa staining for analysis of cellular morphology and phenotyping.

For complete details on the use and execution of this protocol, please refer to Mahony et al.^1^

## Highlights

• Isolation of hematopoietic cells from the adult zebrafish kidney marrow
• Protocol enables total cell counts and analysis of cell populations by flow cytometry
• Steps for preparation of cells for cytospins/cytosmears
• May-Grünwald-Giemsa staining of cells for morphological analysis

## Abstract (editor highlights)

Publisher’s note: Undertaking any experimental protocol requires adherence to local institutional guidelines for laboratory safety and ethics.

## Graphical abstract

**Figure:**
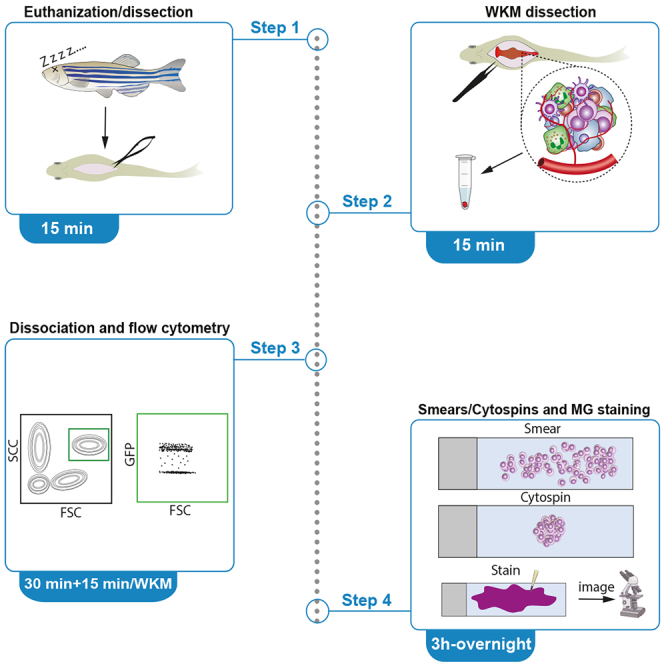

## Before you begin

In zebrafish, Hematopoietic stem and progenitor cells (HSPCs) reside in their adult niche, the kidney marrow. Here they self-renew and differentiate to all blood lineages to sustain hematopoiesis.^2^^,^^3^ Marrow failure happens when HSPCs fail to maintain hematopoiesis, often triggering cytopenias, malignant transformation and eventually leukemia.^4^ To study this process, it is therefore important to characterize the hematopoietic cells in their adult niche. Before you begin, ensure you have sufficient animals of the required genotypes for your analyses and that all the required materials and equipment are ready to use, such as a low magnification stereomicroscope for WKM isolation. See “key resources table” and “materials and equipment” for the lists of these items.

This protocol describes the isolation and analysis of WKM by flow cytometry and cellular phenotyping in fish older than 3 months post fertilization (mpf). However, the same protocol could be applied to younger fish (smaller WKM, thus lower cell numbers per animal) or to isolate cells for analysis by single cell genomics (e.g., scRNA-seq, scATAC-seq).

Before you begin:

1. Ensure you have obtained permission from the relevant institutions regulating animal experimentation before undertaking experiments on adult zebrafish.
2. Seek guidance on local animal regulations for the best practice for zebrafish euthanasia (in this case Schedule 1 killing with Tricaine overdose (5 *g*/L) was used).
3. Obtain training from relevant flow cytometry facility.
4. Grow adult zebrafish of the desired genotypes to 3 mpf or older.
  a. Note that according to new ARRIVE 2.0 guidelines,^5^ animals used should be of both sexes unless your research question requires animals of a specific sex.
5. Prepare Tricaine (5 *g*/L in Tris-HCl buffer, pH 7).
6. Prepare a 10% Fetal Bovine Serum solution in Phosphate Buffer Saline (PBS/10% FBS, pH 7.4).
  a. Prepare and label the required number of Eppendorf tubes to collect individual WKMs and add 500 μL PBS/10% FBS per tube. Keep on ice.

***Note:*** You can either isolate single WKMs or pool several WKMs of the same genotype. For some experiments (*e.g.* sorting enough cells of a specific population), pooling WKMs may be required.

### Institutional permissions

Any experiments on live vertebrates or higher invertebrates must be performed in accordance with relevant institutional and national guidelines and regulations. For this protocol, all experimental procedures in *adult fish were completed in accordance with the Animal Scientific Procedure Act 1986 under an approved Home Office Project License*.

### WKM isolation

**Timing: 15 min per WKM**

**Figure 1:**
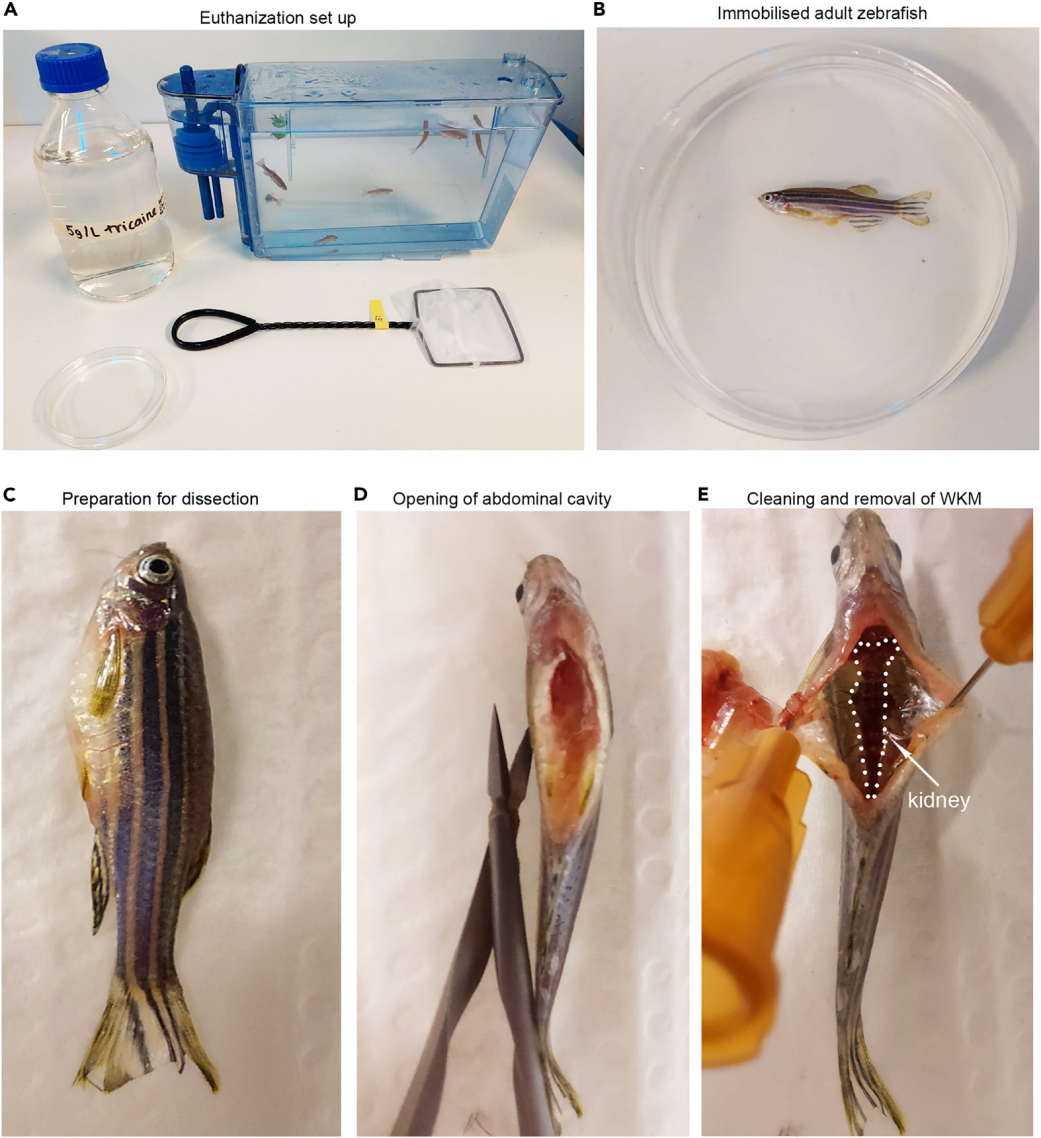
Schedule 1 killing of zebrafish and WKM dissection (A) Euthanization set up and tank of zebrafish. Zebrafish icon by DBCLS https://togotv.dbcls.jp/en/pics.html is licensed under CC-BY 4.0 Unported https://creativecommons.org/licenses/by/4.0/. (B and C) Immobilized fish. (D) Opening of abdominal cavity. (E) Identification and removal of WKM.

7. Euthanize (Schedule 1 killing) adult zebrafish according to local animal regulations.
  a. In this case, zebrafish are placed in buffered Tricaine (5 mg/mL) for approximately 5 min until all movement ceases and the fish is resting on the bottom of the dish (Figures 1A and 1B).
  b. Death was confirmed by removing fish and checking that circulation had ceased by observing the tail fin or the vasculature around the eye on a stereomicroscope.
  c. Fish are placed on their backs on a small piece of sponge (Figure 1C).
  d. Use small scissors to cut the skin from the anus up the belly and to just below the start of the gills (Figure 1D).
  e. Sharp forceps are used to remove all internal organs taking care not to puncture the heart and other vasculature (Figure 1D).
  f. Take extra care to remove eggs from females to avoid contamination.
  g. Use sharp forceps to peel off the WKM from the dorsal wall of the fish (Figure 1E). [optional] Alternatively, a P1000 tip could be used to scrape the WKM.
    **CRITICAL:** Take care not to disrupt the large blood vessel that lies adjacent to the kidney at the back body wall. This will reduce the number of RBCs that may contaminate the sample.
    ***Note:*** This is particularly important when performing RNA-seq, as high hemoglobin levels can skew sequencing and analysis.
    ***Note:*** Sometimes the entire WKM cannot be peeled off completely and several attempts may be required to remove all the cells. Partial collection of the WKM will result in lower cell numbers obtained.
  h. Place the WKM into the prepared Eppendorf with 500 μL PBS/10% FBS and immediately place on ice.
    ***Note:*** For WKM smears, take a small piece of WKM (∼ a third of the total marrow) and follow the instructions in ‘Cytosmears’. Place the remaining two thirds of the WKM into the Eppendorf with 500 μL PBS/10% FBS for flow cytometry analysis.
    ***Optional:*** An entire WKM can be used for a big smear, although for clear results less tissue normally works best.

## Key resources table

**Table:** 

REAGENT or RESOURCE	SOURCE	IDENTIFIER
**Chemicals, peptides, and recombinant proteins**		

Superfrost plus slide	Thermo Scientific	12372098
May-Grünwald	Sigma-Aldrich	MG500
Giemsa stain, modified solution	Sigma-Aldrich	48900-1L-F
DPX	Sigma-Aldrich	44581
FBS	Gibco	26140079
*Hoechst’s dye*	Life Technologies	H3569
10x PBS	Gibco	14200-067
Trypan blue	Sigma-Aldrich	T6146
Tricaine	Sigma-Aldrich	E10521
[optional] Hemocytometer	Marienfeld	
Tris hydrochloride (Tris-HCl)	Merck	10812846001
Cytofunnel	Thermo Scientific	A78710003
5 mL polystyrene filter round bottom tube with filter cap	Falcon	352235

**Experimental models: Organisms/strains**		

Zebrafish: Tg(lck:lck-EGFP)^cz1Tg^. AB strain. Adults (3–12 months), males and females	Langenau et al.^6^	RRID: ZFIN_ZDB-GENO-040723-3
Zebrafish: AB strain. Adults (3–12 months), males and females	N/A	RRID: ZIRC_ZL1

**Software and algorithms**		

AxioVision software	Zeiss	Carl Zeiss Microscopy, LLC - AxioVision Software - Software AxioVision
FACSDiva	BD	BD FACSDiva Software | BD Biosciences

**Other**		

Forceps	WPI	Dumont #5 tweezer inox, 0.01 × 0.05 tips; cat #: 500341
Micro-scissors, straight blade	McPherson Vannas Scissors 8 cm long, straight 5 mm blades, 0.1 mm tips cat #: 14124-G	McPherson Vannas Scissors 8 cm long, straight 5 mm blades, 0.1 mm tips cat #: 14124-G
Stereomicroscope	Nikon	SMZ745t
Microscope with digital camera attachment	Leica	DM750 (microscope) and ICC50W (camera)
Slide scanner	Zeiss	AxioScanner.Z1
Centrifuge	Thermo Fisher Scientific	Cytospin 4 centrifuge

## Step-by-step method details

### Analysis of WKM cells using flow cytometry

**Timing: 30 min + 15 min per WKM**

This step will generate a cell suspension from dissected WKM tissue that will be analyzed using flow cytometry to examine the proportions of the major cell populations present.

**Figure 2:**
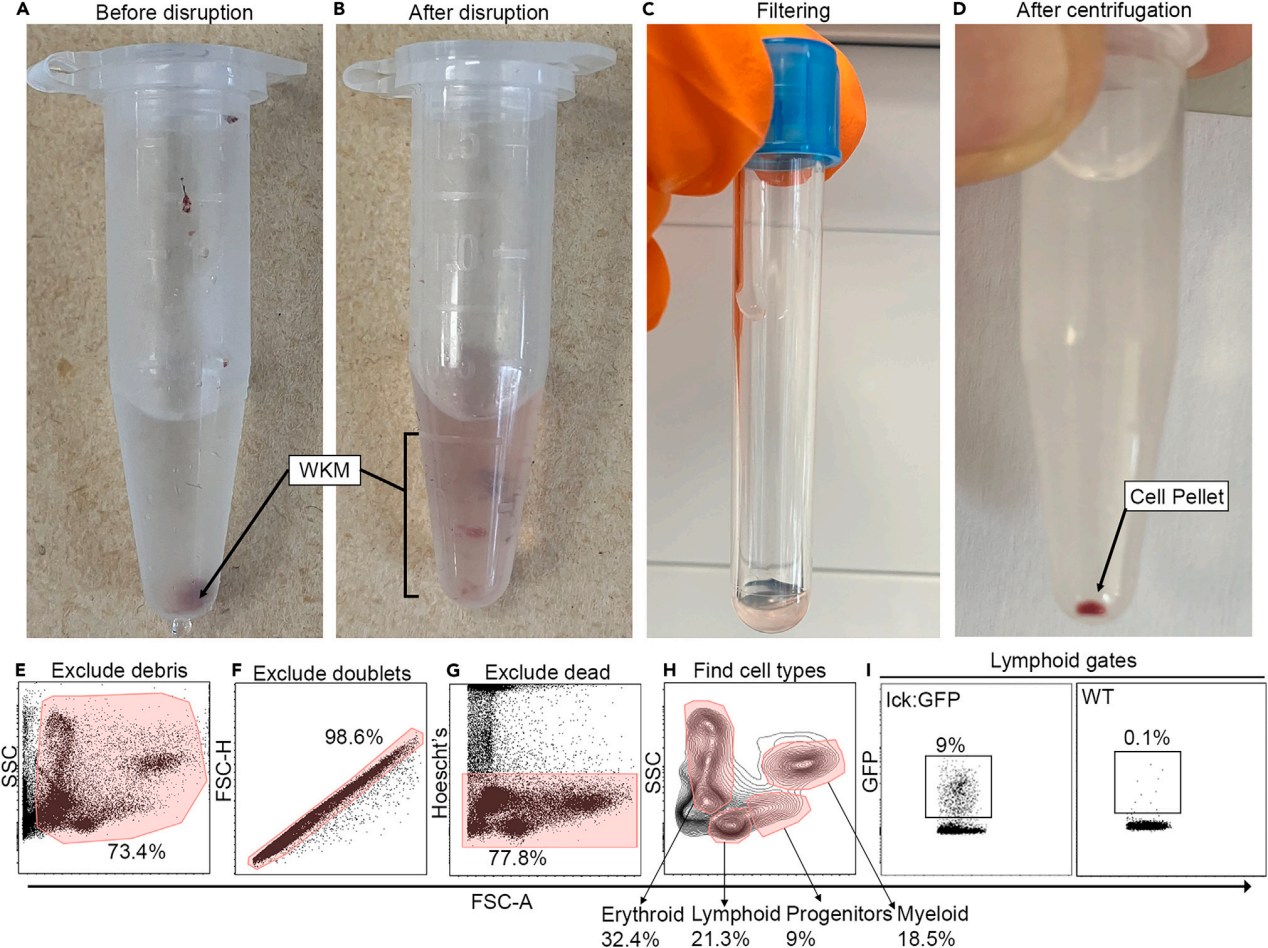
Preparing single cell suspension of WKM for flow cytometry analysis (A and B) Before and after dissociation of WKM. (C) Filtering of WKM. (D) Centrifuging and identification of cell pellet. (E‒I) Gating strategy for analysis.

1. WKM dissociation.
  **CRITICAL:** Select a P1000 **non-filter** pipette and pipette tip. Cells will generally get trapped in a filter resulting in loss of cells.
  a. Apply the pipette tip into the PBS/10% FBS solution (use ∼500 μL) with WKM and pipette up/down 5–10 times until the liquid becomes cloudy (Figures 2A and 2B).
    ***Note:*** Insufficient pipetting will lead to lower cell recovery due to incomplete tissue dissociation. By contrast, too much pipetting will likely lead to increased apoptosis.
  b. Repeat additional times should there still be a large piece of WKM remaining.
    ***Note:*** Some kidney tissue will always remain, this is normal.
2. Filtering cells.
  a. Apply around 100 μL of PBS/10% FBS to a clean and dry 5 mL polystyrene filter round bottom tube with filter cap.
    **CRITICAL:** The filter (35 μm) must not be dry before applying sample.
  b. Add the sample to the tube (∼500 μL).
  c. Allow the sample to drip through the filter (Figure 2C).
  d. Apply 500 μL PBS/10% FBS to the filter once the sample has drained.
  e. Repeat step d. three more times.
  f. Spin sample at 1000 rpm (∼300 *g*) for 5 min at 4°C. A red pellet should be readily visible (red blood cells, Figure 2D). This might not be present on smaller WKMs or samples with fewer cells.
  g. Remove supernatant and resuspend cells in 200 μL PBS/10% FBS+ Hoechst for live/dead cell discrimination during flow cytometry (dilute Hoechst 1:10000 in PBS/10% FBS).
    ***Note:*** This solution can be prepared earlier (e.g. between steps c. and e.)
  h. Place on ice in the dark until ready for analysis.
3. Flow cytometric analysis (Figures 2E–2H).
  a. [optional] Cells can be counted using a hemocytometer and dead cells stained using trypan blue (generally diluted 1:10) and the total number of cells can be calculated.
    ***Note:*** This can be used to infer the total number of cells from percentages following analysis of flow data. For example, if we take the approximate average number of live cells in a wild-type 6 mpf WKM (1.4 × 10^5^ cells, see Figure 1B from^1^) we can infer typical cell numbers (from the example in this study, see Figure 2G: lymphoid: 29,820 cells, progenitors: 12,600 cells, myeloid: 25,900 cells, erythroid: 45,360).
  b. Bring samples to flow cytometer. In this case a *BD LSRFORTESSA X-20 and* BD FACSDiva *software were used.*
  c. Ensure Fortessa is switched on, lasers have warmed up (this will take ∼20 min) and BD FACSDiva has been opened.
  d. Perform necessary cleaning steps on Fortessa.
  e. Ensure lasers are switched on to detect Hoechst (in this case BV510) and any other fluorescent marker being investigated.
    **CRITICAL:** Run the first sample and exclude debris. Erythrocytes have low FSC and therefore sit closely to debris.
    ***Note:*** In addition to this, the FSC on a BD Aria (and possibly other machines) does not discriminate erythrocytes. Care must be taken to not include debris in the analysis of these cells as much as possible (Figure 2E).
  f. Then doublets can be excluded in different ways, such as plotting FSC-area vs. FSC-height. The area of doublets will be much higher than single cells, while the height will be similar to single cells. Cells with disproportionate area/height can therefore be excluded (Figure 2F).
  g. Exclude dead cells by gating on Hoechst low/negative cells (Figure 2G).
  h. Set up FSC on x axis (linear scale) and SSC on y axis (log scale) (Figure 2H).
    **CRITICAL:** Adjust FSC and SCC laser setting so that the majority of cells are on scale. These adjustments will vary depending upon make/model of machine used and individual setups.
  i. Run enough cells to observe the four main populations (erythrocytes, myeloid cells, progenitors and lymphoid cells) and adjust gates accordingly (Figure 2H). This is typically anywhere between 10 k and 50 k cells.
  j. Optional: Fluorescently labeled cells can be measured within the populations (Figure 2I).
    **CRITICAL:** If multiple colors are being used, you will require a ‘fluorescence minus one’ sample whereby one of the colors being examined is not present. This will establish the threshold for negative cells for the absent color.
  k. Optional: fluorescently labeled cells can be isolated using FACS (e.g., **BD FACSAria Fusion**) for further processing (e.g., scRNA-seq, scATAC-seq).

### Cytospins

**Timing: 20 min – this will take longer for more WKMs**

This step will enable the deposition of unsorted or sorted WKM cell populations on a slide to allow the staining of hematopoietic cells and evaluation of cell numbers and cell morphology.

**Figure 3:**
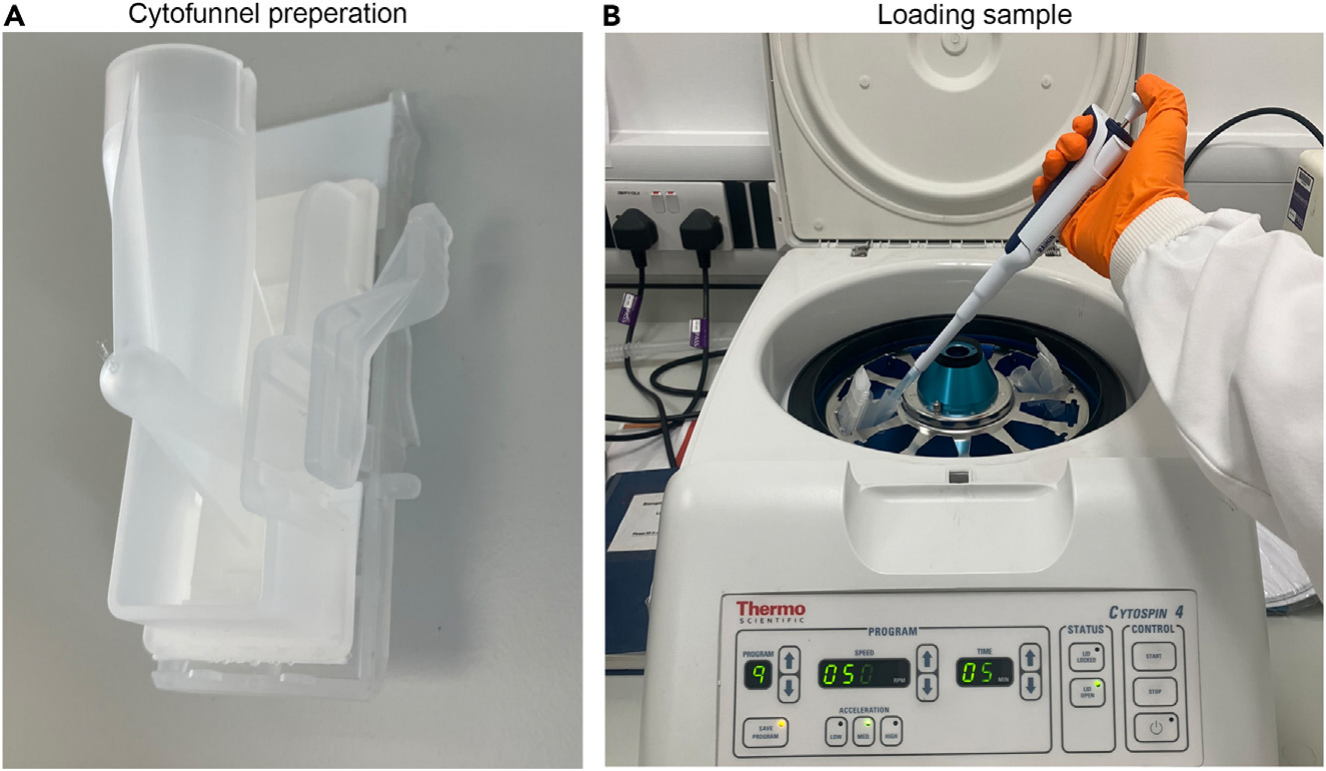
Cytospining cells (A) loading slide into cytofunnel. (B) Loading sample into cytofunnel in cytospin centrifuge.
  b. Place cytofunnel in the Cytospin centrifuge.
  c. Apply cell suspension to the cytospin funnel (no more than 200 μL) (Figure 3B).
  d. Spin at 500 rpm (∼29 *g*) for 5 min.
  e. Carefully remove the slide and air dry for no more than 20 min.
  f. Be careful not to disturb the cells.
  g. Proceed directly to ‘Cytosmears’.

4. An aliquot of cells isolated from a WKM (or an entire WKM) can be resuspended in PBS/10% FBS and cytospun for May-Grünwald-Giemsa staining and morphological analysis.
  a. Place a Superfrost plus slide into a cytofunnel (Figure 3A).

### Ctyosmears

**Timing: 1 h**

This step provides instructions to smear WKM cells on slides following dissection allowing staining and visualization of the major cell types and morphologies. Cell morphologies tend to be better preserved in cytosmears compared to cytospun samples.

**Figure 4:**
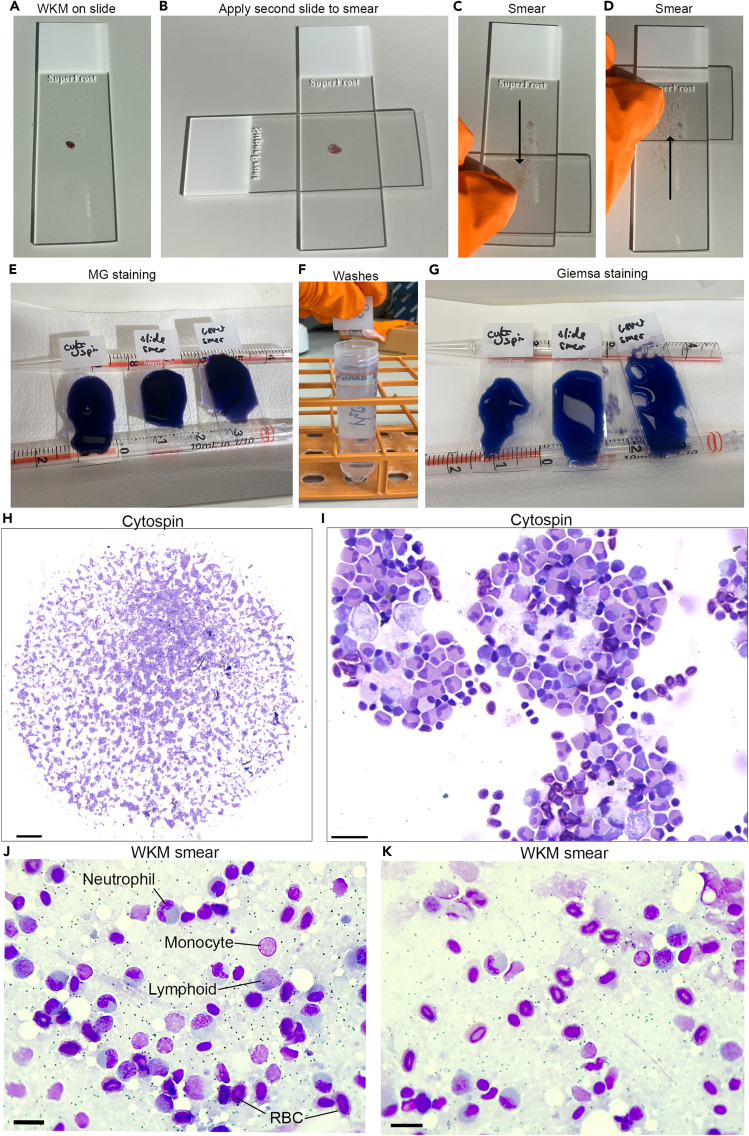
WKM smears and staining cells (A‒D) Smearing WKM on slide. (E‒G) MG staining. (H and I) Cytospun cells. (J and K) WKM smears. Scale bars: H- 500 μm, I- 20 μm, J and K- 10 μm.
  b. Air-dry the slide for at least 20 min at 18°C–22°C.

5. Place a freshly dissected WKM piece (around one third of total WKM) on a Superfrost plus slide.
  a. Place a dry and clean cover slip over the WKM piece and push to smear the tissue across the slide (Figures 4A–4D).

**CRITICAL:** Proceed immediately to step 6, do not leave slide to air dry for more than 2 h.

### May-Grünwald-Giemsa (MGG) staining

**Timing: 1–16 h**

This step will stain WKM smears or cytospun WKM cells with May-Grünwald-Giemsa (MGG) stain to allow differential staining of the different blood cell types and analysis of cell morphologies.

6. This protocol will use slides with cytospun cells from **4.** or smeared cells from **5.** once air drying is completed.
  a. Dilute May-Grünwald (MG) stain 1:1 with distilled water and Giemsa 1:10 with distilled water.
  b. Incubate slides with diluted MG for 5 min at 18°C–22°C (Figure 4E).
  c. Wash slides with distilled water (5–10 times up/down) (Figure 4F).
  d. Incubate slides diluted Giemsa for 30 min at 18°C–22°C (Figure 4G).
  e. Wash slides with distilled water (5–10 times up/down) and then air dry.
    **CRITICAL:** slides must be completely dry before proceeding.
  f. Slides are then mounted using a small volume of DPX and left to set for around 16 h.
  g. Image as required, this could be completed using slide scanner (Figures 4H and 4I) or microscope with digital attachment (Figures 4J and 4K).

## Expected outcomes

This protocol will enable the user to isolate WKM from adult zebrafish and create a WKM smear which can be used for MGG staining to identify the main cell populations and cell morphologies (Figures 4J and 4K). Alternatively, the isolated WKM can be used to create a cell suspension for flow cytometry analysis and quantification of the main hematopoietic populations (Figures 2E–2H), or further morphological analysis of specific populations after cytospin and MGG staining (Figures 4H and 4I). Finally, the WKM cell suspension can be processed for single cell genomics applications such as scRNA-seq or scATAC-seq.

## Limitations

The number of cells obtained (especially from younger fish, or from samples with fewer cells dues to e.g., marrow failure) will limit the number of different assays that can be used to analyze a single WKM. This can sometime be overcome by pooling tissue. A major limitation in zebrafish biology is the lack of reliable antibodies to stain for intracellular/surface markers for flow cytometry analysis. Most labs resort to using transgenic lines which can limit analysis. May-Grünwald-Giemsa staining robustly stains cells but unbiased machine learning based analysis requires access to specialist software trained to zebrafish cells to perform analysis of all cells in the smear/cytospin. Therefore, combining these techniques is important to accurately assess the WKM cells.

## Troubleshooting

### Problem 1

Yield of cells is too low for analysis after step 1.

### Potential solution

Do not use filter tips for 2a and ensure that pipetting is not too forceful that liquid will be lost from the Eppendorf in 3c.

### Problem 2

Sample does not drain through the filter in step 3.

### Potential solution

Gently tap the side of the tube to encourage liquid to filter through.

### Problem 3

Cytospun sample cells have a damaged appearance after step 5.

### Potential solution

The cells will not look the same as smeared cells (Figures 4J and 4K); they will have a slightly damaged appearance due to the centrifugation step. But if they appear to be lysed then reduce centrifuge speed.

### Problem 4

Smeared WKM cells are too clumped after step 5.

### Potential solution

The often happens when smearing too large a piece of WKM, reducing the size of the piece can help this. Repeat the smear in 5a immediately after the first smear ensuring the tissue is evenly spread across the slide.

### Problem 5

May-Grünwald-Giemsa staining has too much background after step 6.

### Potential solution

Increase the time and number of distilled water wash steps (step 6c). Ensure clean water is used.

### Problem 6

Flow cytometry analysis shows that the majority of cells are dead (i.e., high positive fraction for live/dead stain) after step 4.

### Potential solution

Decrease the number of times WKM is pipetted up/down. Keep cells on ice during incubation steps. Remake reagents, check FBS is sterile and fresh. Keep time in between steps to a minimum. Ensure live/dead dye is added just before flow cytometry analysis (to prevent over labeling of cells).

## Resource availability

### Lead contact

Further information and requests for resources and reagents should be directed to and will be fulfilled by the lead contact, Rui Monteiro (r.monteiro@bham.ac.uk).

### Technical contact

Further information and requests for technical advice should be directed to and will be fulfilled by the technical contact, Christopher B. Mahony (c.mahony@bham.ac.uk)

### Materials availability

This study did not generate new unique reagents.

### Data and code availability

This study did not generate/analyze datasets/code.
